# Automated quantification of steatosis: agreement with stereological point counting

**DOI:** 10.1186/s13000-017-0671-y

**Published:** 2017-11-13

**Authors:** André Homeyer, Patrik Nasr, Christiane Engel, Stergios Kechagias, Peter Lundberg, Mattias Ekstedt, Henning Kost, Nick Weiss, Tim Palmer, Horst Karl Hahn, Darren Treanor, Claes Lundström

**Affiliations:** 1 Fraunhofer MEVIS, Am Fallturm 1, 28359 Bremen, Germany; 2 Center for Medical Image Science and Visualization, Linköping University, 581 83 Linköping, Sweden; 3 Department of Medical and Health Sciences, Linköping University, 581 83 Linköping, Sweden; 4 Department of Radiation Physics, Linköping University, 581 83 Linköping, Sweden; 5 Institute of Cancer and Pathology, University of Leeds, Beckett Street, Leeds, LS9 7TF UK; 6 Leeds Teaching Hospitals NHS Trust, Beckett Street, Leeds, LS9 7TF UK

**Keywords:** Steatosis, Histology, Stereology, Stereological point counting, Automated image analysis, Agreement

## Abstract

**Background:**

Steatosis is routinely assessed histologically in clinical practice and research. Automated image analysis can reduce the effort of quantifying steatosis. Since reproducibility is essential for practical use, we have evaluated different analysis methods in terms of their agreement with stereological point counting (SPC) performed by a hepatologist.

**Methods:**

The evaluation was based on a large and representative data set of 970 histological images from human patients with different liver diseases. Three of the evaluated methods were built on previously published approaches. One method incorporated a new approach to improve the robustness to image variability.

**Results:**

The new method showed the strongest agreement with the expert. At 20× resolution, it reproduced steatosis area fractions with a mean absolute error of 0.011 for absent or mild steatosis and 0.036 for moderate or severe steatosis. At 10× resolution, it was more accurate than and twice as fast as all other methods at 20× resolution. When compared with SPC performed by two additional human observers, its error was substantially lower than one and only slightly above the other observer.

**Conclusions:**

The results suggest that the new method can be a suitable automated replacement for SPC. Before further improvements can be verified, it is necessary to thoroughly assess the variability of SPC between human observers.

## Background

Hepatic steatosis denotes the excessive accumulation of fat in the liver. It can be induced by different causes, including alcohol misuse, obesity, or drug toxicity [1]. Steatosis is the hallmark of fatty liver disease (FLD), the most frequent liver disorder in Western countries [2]. Depending on the primary cause, it is common to distinguish between alcoholic (AFLD) and nonalcoholic fatty liver disease (NAFLD). Both share similar histological features and, if left untreated, can progress into steatohepatitis, cirrhosis, hepatocelluar carcinoma and liver failure [1, 3]. NAFLD is expected to become one of the most common indications for liver transplantation in the world [4].

The gold standard for the assessment of steatosis is the visual analysis of histological sections taken from liver biopsies or resections. Histological analysis of steatosis is performed routinely to diagnose FLD or to decide about the suitability of liver grafts for transplantation [5]. In addition, it plays a vital role in research, and tissue sections are commonly analyzed for steatosis to understand the cause and to improve the treatment of liver diseases. Other research applications include the assessment of drug toxicity, or the validation of non-invasive means for analyzing steatosis [6].

**Fig. 1:**
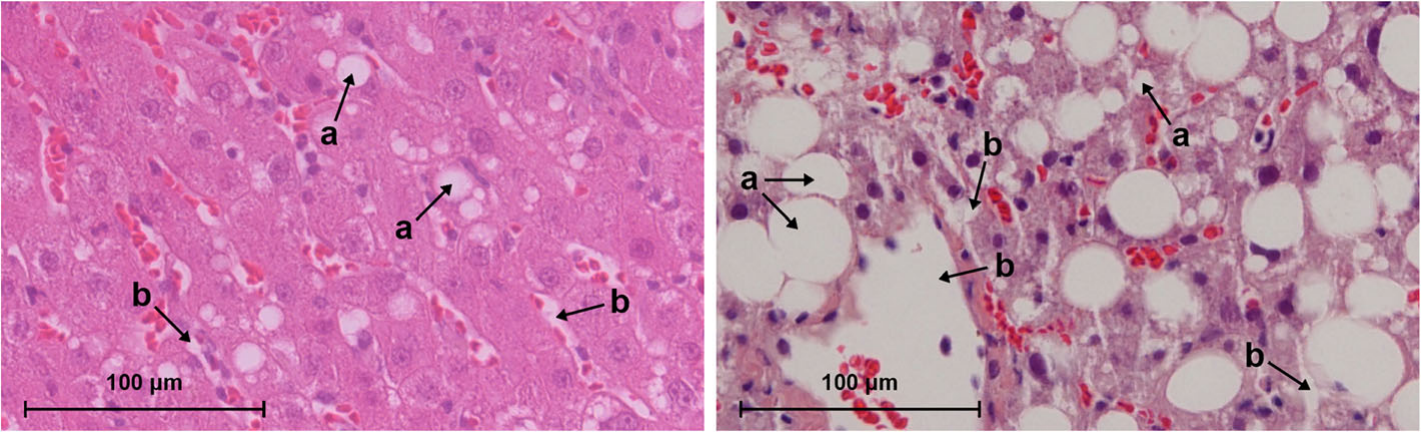
Histological appearance of steatosis. Low-grade and high-grade steatosis are shown left and right, respectively. Fat droplets (a) and other empty spaces (b) are marked by arrows

Steatosis is usually assessed in paraffin-embedded, Hematoxylin and Eosin-stained (HE) sections, where it is visible as fat droplets in the cytoplasm of hepatocytes. Since fat is dissolved during histological processing, fat droplets appear as empty spaces in the tissue. Fat droplets can be distinguished from other empty spaces, like vessels or tissue cracks, by their size and roundish shape (see Fig. 1).

When cells are occupied by one large fat droplet, which displaces the nucleus to the periphery, or multiple small fat droplets one speaks of macrovesicular steatosis. When cells are filled by innumerable tiny fat droplets, that make the cytoplasm appear “foamy”, one speaks of microvesicular steatosis [7, 8]. In this paper, we only consider macrovesicular steatosis, which is most common and of main interest in the aforementioned applications [5, 7]. This includes small-droplet macrovesicular steatosis, which is often erroneously referred to as microvesicular steatosis [9].

It is standard practice to quantify macrovesicular steatosis in terms of the proportion of hepatocytes that contain fat droplets. The result is expressed as a semi-quantitative grade from 0 to 3 (0 is <5%; 1 is 5%–33%; 2 is 34%–66%; 3 is >66%) [7]. A major problem of the cell-based quantification is that, since individual cells are not clearly delineated, the association between fat droplets and cells is often arbitrary. Also, to save time, the number of relevant cells is usually not counted, but merely estimated. Both are reasons why the cell-based estimation of steatosis is often only poorly reproducible [10, 11].

**Fig. 2:**
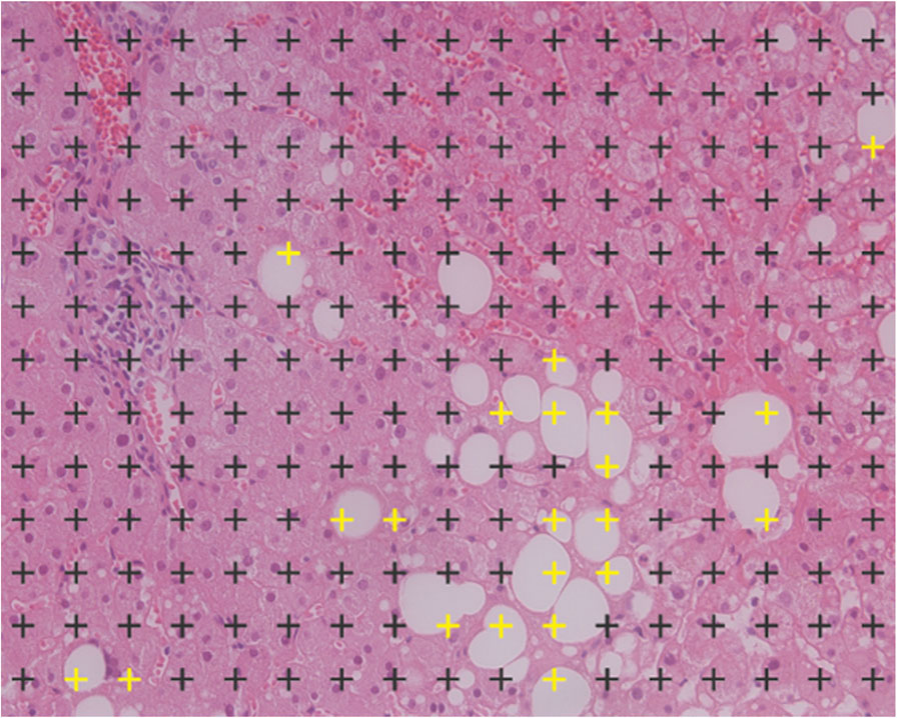
Stereological point counting. A region of interest is overlaid by a regular grid of points that are counted as fat (yellow) or no-fat (gray)

Stereological point counting (SPC) is an alternative method for quantifying steatosis in histological sections. In SPC, a region of interest is overlaid by a regular grid of points. The points are counted as fat or no-fat depending on whether they cover fat droplets or not (see Fig. 2). The number of points covering fat droplets divided by the total number of points yields an estimate of the area fraction of fat droplets. It has been shown, that the area-based quantification of steatosis by means of SPC is highly reproducible [11–13]. However, since it is much more time-consuming than the cell-based estimation, it is only rarely performed in practice.

Automated image analysis can greatly reduce the quantification effort. There are many previous publications on image analysis methods for quantifying steatosis. In most of them, the presented methods are evaluated against other means for quantifying steatosis. It is common to assess the correlation with cell-based visual estimates by human observers. The correlation is found to be strong in some publications [14, 15] and weak in others [10, 16]. Many publications also assess the correlation with biochemical analyses of the tissue fat content. Here, the correlation is generally found to be strong [12, 15–17].

For practical applications, it is essential that different analysis methods agree on measured steatosis values. Only then it is possible to define clinical diagnostic thresholds or to compare research results. Virtually all previous publications have only evaluated correlations with other measurement methods. Correlation, however, does not necessarily imply agreement [18].

Automated image analysis methods usually quantify steatosis in terms of the area fraction of fat droplets. This makes it impossible to assess agreement with cell-based visual estimates or biochemical analyses, because the methods measure different quantities [16]. For the assessment of agreement, image analysis methods must be evaluated against other area-based methods of measurement, such as SPC. However, due to its high effort, SPC-based measurements are only rarely available.

Sciarabba et al. [19] appears to be the only publication that has evaluated the agreement between automated image analysis and SPC. While the agreement is found to be high, the results have limited generalizability because the evaluation was based on only a single analysis method and four biopsies.

We have performed a comprehensive evaluation of different image analysis methods in terms of their agreement with SPC performed by a hepatologist. The evaluation was based on a large and representative data set of 970 images from human patients with different liver diseases. As described in the next section, we have evaluated three methods built on previously published approaches and one new method. To assess its practical utility, we have compared the best performing method with SPC by two additional human observers.

## Methods

### Data

The evaluation was based on histological images of liver biopsies of human patients. The images were supposed to be representative for clinical practice, both in terms of quality and variability.

Liver biopsies were obtained from patients referred to the Department of Gastroenterology and Hepatology at Linköping University Hospital, Linköping, Sweden, for evaluation of chronically (≥ 6 months) elevated levels of serum alanine aminotransferase (ALT; elevated defined as >71 U/L for men and >45 U/L for women) and/or aspartate aminotransferase (AST; elevated defined as >45 U/L for men and >36 U/L for women) and/or serum alkaline phosphatase (ALP; defined as >106 U/L for both sexes). A diagnostic work-up was performed and all patients who, on clinical indication, needed a liver biopsy for diagnosis where asked to participate in our study.

The patients included in the study suffered from different liver diseases, such as non-alcoholic fatty liver disease (NAFLD), autoimmune hepatitis (AIH), and hepatitis C virus infection (HCV). The biopsies, therefore, exhibited different degrees of steatosis, inflammation and fibrosis.

In total, 97 HE-stained slides were considered, with each slide containing liver tissue from a different patient. Exactly 10 field-of-view images were captured from every slide, each measuring 0.59 mm × 0.47 mm. To be representative for clinical practice, the images were captured using common laboratory equipment, consisting of a Nikon Eclipse E800 microscope and Nikon DS-Ri1 digital camera.

All images were resampled to three resolutions. For simplicity, we refer to each resolution by the magnification of an objective lens that produces a similar visual impression [20]. Table 1 lists the different resolutions and the corresponding magnifications and image sizes.

**Table 1:** Image resolutions and sizes

Magnification	Resolution	Image size
5×	1.84 μm/pixel	320 × 256
10×	0.92 μm/pixel	640 × 512
20×	0.46 μm/pixel	1280 × 1024

In order to minimize selection bias, the positions of the images within the slides were chosen according to a system described by Franzén et al. [11]. The first image was positioned at the outermost end of the biopsy. The following images were positioned by iteratively moving 1.25 times the size of the field of view along the direction of the biopsy. In this manner any overlap between individual fields of view was prevented.

The slides were randomly divided into disjoint training and test sets. The images from the training slides were used for training the analysis methods. The images from the test slides were used for evaluating the resulting analysis performance. The training and test sets contained 25 and 72 slides, respectively, resulting in 250 training and 720 test images.

### Training set

In the 250 training images, examples of relevant image structures were annotated for the training of machine-learning classifiers in the analysis methods. The annotation was performed by a computer scientist experienced in histological image analysis.

Two sets of annotations were created. The first set, called “pixel samples”, consisted of examples of foreground pixels and background pixels. Foreground pixels represent empty spaces, such as fat droplets, vessels, tissue cracks or background. Background pixels represent stained tissue. Approximately 6000 examples of each class where annotated in total. Examples were selected with the objective of covering the variability of colors as much as possible, in particular, with regard to border regions between fat droplets and stained tissue.

The second set, called “blob samples”, consisted of examples of fat droplets and other empty spaces, such as vessels or tissue cracks. Approximately 500 examples of each class were annotated. Examples were selected with the objective of covering the variability of sizes and shapes as much as possible.

### Test set

The degrees of steatosis in the 720 test images were estimated using SPC. The procedure was performed independently by three human observers. The first counting was performed by a resident hepatologist (Observer 1) and validated by another hepatologist with more than 14 years of experience in the morphometric quantification of hepatic steatosis [6]. The second and third countings were performed by a medical technical assistant (Observer 2) and a computer scientist (Observer 3), who were knowledgeable in the task and experienced in the quantitative analysis of medical images.

SPC was performed as described by Franzén et al. [11]. Every image was overlaid by a grid of 221 (17 × 13) points with an equal spacing of 35 μm, so that most of the larger vacuoles were covered by at least one point (see Fig. 2). No distinction was being made between small and large fat droplets. Hepatocytes in which individual fat droplets could not be clearly distinguished were omitted.

El-Badry et al. recommend to perform the evaluation separately for low-grade and high-grade cases [10]. Since low-grade steatosis covers only a small value range, its acceptable error is smaller than that for high-grade steatosis. In this regard, we have divided the test images into a low-grade and high-grade set guided by semiquantitative grades given by a liver pathologist. Absent or mild steatosis (grade 0 or 1) was considered to be low-grade, while moderate or severe steatosis (grade 2 or 3) was considered to be high-grade [10].

### Analysis methods

We have implemented four different automated image analysis methods for quantifying steatosis in order to compare their agreement with SPC.

**Fig. 3:**
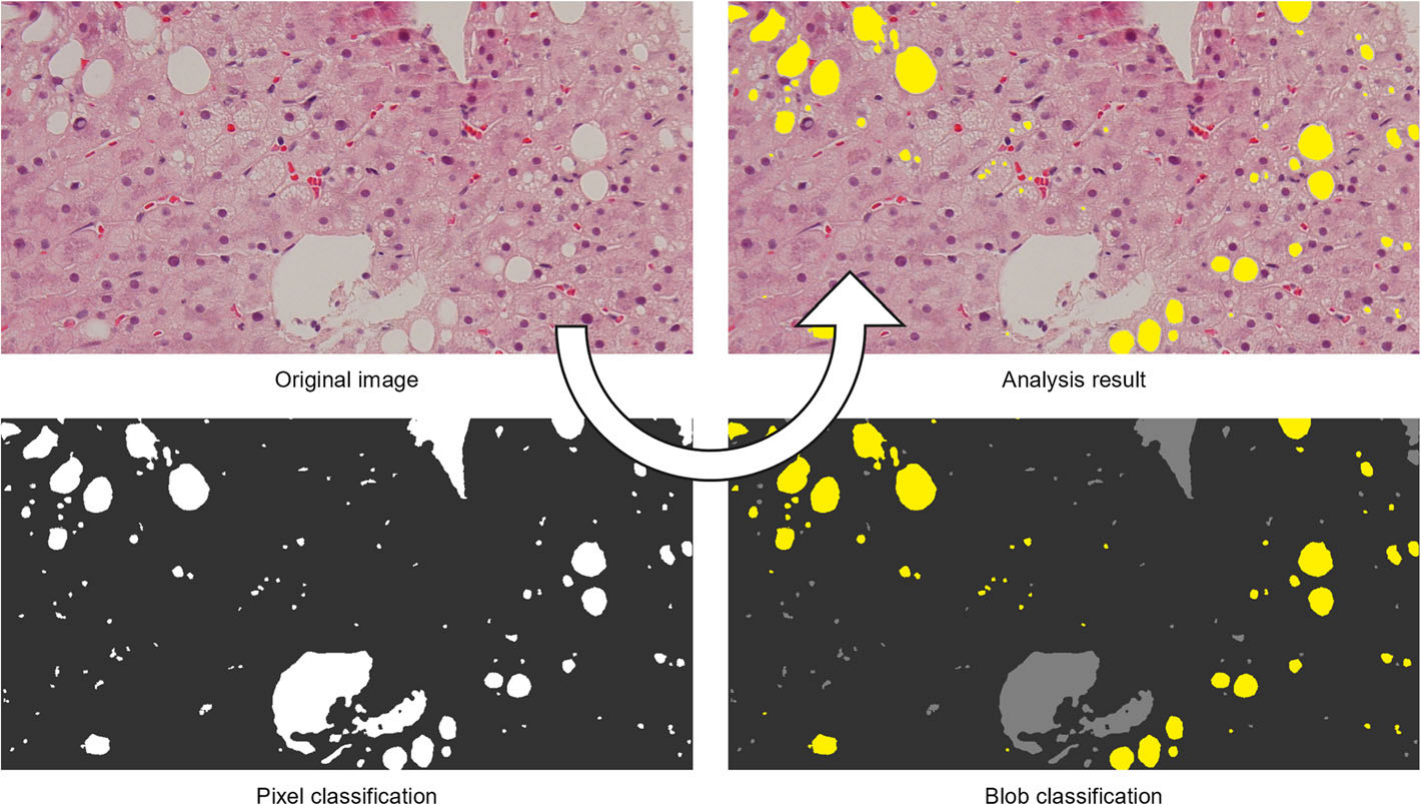
Overview of the analysis framework. First, pixels are classified as foreground or background. Afterwards, blobs of foreground pixels are classified as fat or other empty spaces

All methods were based on a commonly employed, unified framework of two successive pixel and blob classification steps (see Fig. 3). In the pixel classification step, pixels are classified as foreground or background. Foreground pixels represent bright empty spaces in the tissue, background pixels represent the stained tissue itself. In the blob classification step, connected areas of foreground pixels, called “blobs”, are classified as fat or other empty spaces, such as vessels or tissue cracks.

The result is a map of the same size as the original image in which pixels are labeled as either fat or no-fat. The final steatosis score is computed as the relative fraction of pixels labeled as fat.

For evaluation purposes, an additional SPC estimate of the steatosis score is computed. This estimate is obtained by sampling the map on the same grid as used by the human observers, and then computing the relative fraction of grid points labeled as fat. For brevity, we call pixel-based steatosis scores “pixel scores” and point-based steatosis scores “point scores”.

The four methods differ in their implementation of the pixel and blob classification steps as described below. The first three methods are built on previously published approaches. The fourth method incorporates a new pixel classification approach. Methods 1–4 become successively more complex and computationally expensive. All methods were implemented in C++ and run on an Intel Xeon CPU E5430 with 8 GB working memory.

### Pixel classification

#### Methods 1–3

Empty spaces are generally brighter and less saturated than tissue areas. It is therefore common practice to classify pixels by fixed thresholds to color values [12, 15, 17, 21–24] and to green channel values in particular [10, 14, 16]. Methods 1–3 employ a Random Forest Classifier (5 trees) [25] for the pixel classification that automatically derives appropriate green channel thresholds from the pixel samples in the training set.

#### Method 4

**Fig. 4:**
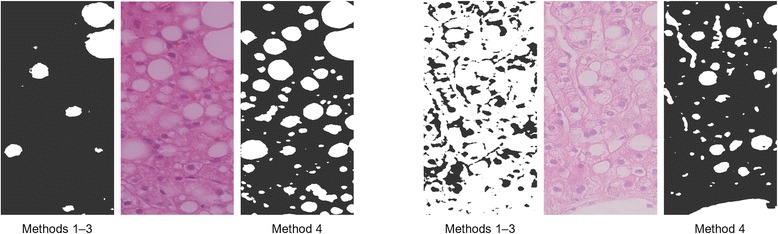
Robustness to image variability. Method 4 incorporates a new pixel classification approach to improve the robustness to image variability

Histological images often vary so much that empty spaces in one image appear darker or more saturated than tissue areas in another image. In this case, the application of fixed thresholds to color values produces erroneous results. Method 4 incorporates a new pixel classification approach that intends to be more robust to such variability (see Fig. 4). This approach is based on two common image processing operations and, therefore, simple to implement in software.

Instead of considering absolute pixel values, it makes assumptions about relative pixel values with respect to their surroundings. The first assumption is that foreground pixels are generally less saturated than their surrounding tissue area. In this respect, the classification considers the difference of the saturation of individual pixels and the mean saturation in a radius of ~60 μm (128 pixels at 20× resolution).

The second assumption is that brightness values within fat droplets are fairly constant. In this respect, the classification considers the local derivative of the brightness of individual pixels, computed with the 3 × 3 Sobel operator. Again, a Random Forest classifier (5 trees) is employed for the evaluation of both features that was trained on the pixel samples in the training set.

### Blob classification

#### Method 1

For simplicity, Method 1 assumes all blobs to be fat droplets. This approach is typically used when no specialized software for quantifying steatosis is available [12].

#### Method 2

Fat droplets tend to be differently sized and less elongated than other empty spaces, such as vessels or tissue cracks. It is, therefore, common practice to classify blobs by shape features that quantify their size and their eccentricity or roundness [14–16, 21–23]. Method 2 classifies blobs by their number of pixels and eccentricity. The classification itself is performed by a Random Forest classifier (21 trees) that was trained on the blob samples in the training set.

#### Methods 3 & 4

Squeezed or clustered fat droplets, compact artifacts or cross-sectioned vessels can assume all kinds of shapes. In these cases, fat droplets and other empty spaces cannot be distinguished by size and eccentricity features alone. In Homeyer et al. [26], adjacency statistics features were shown to improve the distinction of complex blob shapes. For this reason, Method 3 & 4 add adjacency statistics to the size and eccentricity features used in Method 2. The classification itself is, again, performed by a Random Forest classifier (21 trees) that was trained on the blob samples in the training set.

### Evaluation of agreement

We compare different image analysis methods in terms of their error against SPC performed by a human observer. In addition to mean absolute error values, we present success rate curves and Bland-Altman plots. The results are presented separately for low-grade and high-grade images.

For practical applicability, any new measurement method must produce a smaller error than an acceptable maximum error in a minimum percentage of cases. Two inverse metrics are often considered in this regard: the coverage probability (CP) and the total deviation index (TDI) [27]. The CP describes the percentage of cases for which the error is smaller than a given acceptable maximum error. The TDI describes the maximum error of a given minimum percentage of cases.

**Fig. 5:**
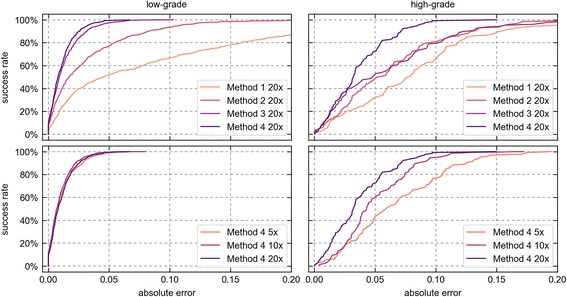
Success rate curves of different analysis methods. The x-axes show absolute error levels (in area fraction), the y-axes give the corresponding percentage of images on which the absolute error was below or equal to that level

There is no general definition of the acceptable maximum error or the minimum percentage of cases for the quantification of steatosis. For this reason, we present plots where different TDIs are plotted on the x-axis against their respective CPs on the y-axis (see Fig. 5). Such plots depict the cumulative distribution of absolute errors over all images. They are also called “success rate curves” because they plot the rates of cases that succeed in satisfying a certain quality criterion (the maximum acceptable error). Success rate curves enable the intuitive comparison of the error of multiple measurement methods. The superiority of one analysis method over another is proportional to how much its success rate curve lies left of the one of the other method.

Bland-Altman plots are another common way for assessing agreement between two quantitative measurement methods [18]. Bland-Altman plots visualize combined measurements as dots. The mean of two measurements is plotted on the x-axis against their difference on the y-axis. For easier interpretability, the median of all differences is drawn as a horizontal line. Two further horizontal lines are drawn at the 2.5th and 97.5th percentiles, signifying the limits of agreement. If normality can be assumed, the lines are drawn at the mean of the differences and ±1.96 times the standard deviation, respectively. Bland-Altman plots make it simple to assess whether there is any systematic bias in the measurements and how the error relates to their magnitude.

## Results

### Method evaluation

We have evaluated all four analysis methods in terms of their error against SPC by Observer 1. For this, all analysis methods were trained and tested at 20× resolution. The evaluation was based on point scores, so that the sampling error of SPC could be ignored. Since the practical value of any analysis method depends on both its error and its runtime, we have measured the mean runtime per image of all analysis methods. The results are summarized in Table 2 and Fig. 5 (top row).

**Table 2:** Method evaluation results

Method	Mean Absolute Error low-grade	Mean Absolute Error high-grade	Mean Runtime
Method 1 20×	0.082 (±0.087)	0.083 (±0.059)	1.01 (±0.17)
Method 2 20×	0.032 (±0.037)	0.062 (±0.048)	1.01 (±0.17)
Method 3 20×	0.013 (±0.014)	0.060 (±0.052)	1.02 (±0.17)
Method 4 5×	0.011 (±0.012)	0.067 (±0.041)	0.15 (±0.01)
Method 4 10×	0.010 (±0.010)	0.050 (±0.029)	0.51 (±0.02)
Method 4 20×	0.011 (±0.011)	0.036 (±0.026)	1.90 (±0.08)

Mean absolute errors are given in area fraction and mean runtimes per image are given in seconds (± std. dev)

The simple threshold-based segmentation of Method 1 generally performed worst. Obviously, sorting out vessels or tissue cracks is essential for accurate results. While Method 2 and 3 performed similarly on high-grade images, Method 3 performed significantly better than Method 2 on low-grade images. Here, its utilization of adjacency statistics caused fewer vessels or tissue cracks to be misclassified as fat droplets.

The best results on both image sets were produced by Method 4. Its new pixel classification approach improved the robustness to variability in the color values (see Fig. 4), which had a substantial effect on the error of high-grade images. However, the superior accuracy came at the cost of computation time. While the mean runtimes of Methods 1–3 were virtually the same, the computation of two extra feature channels in Method 4 increased the runtime almost twofold.

The image resolution is a major factor in the runtime of analysis methods. We have, therefore, additionally evaluated two variants of Method 4 that were trained and tested at 10× or 5× resolution. The results are summarized in Table 2 and Fig. 5 (bottom row).

While all variants performed similarly on low-grade images, the error increased substantially from 20× to 10× and from 10× to 5× on high-grade images. At higher resolutions, the considered shape features proved to be more discriminative for the complex blob shapes encountered in high-grade images.

Since the pixel classification is the main contributor to the computational costs, the mean runtime of Method 4, along with the number of pixels in the image, was divided by approx. four from 20× to 10× and from 10× to 5× resolution.

The error of Method 4 at 10× was still lower than the error of Method 3 at 20×. At the same time, the average execution of Method 4 at 10× took only half as long as Method 3 at 20× .

### Observer evaluation

**Fig. 6:**
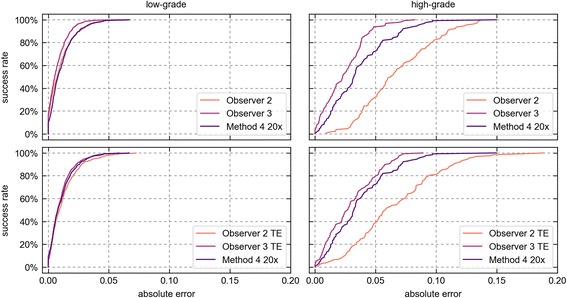
Success rate curves of Observer 2 & 3 and Method 4. The top row compares the inherent error of Observer 2 & 3 and Method 4. The bottom row compares the estimated total error of Observer 2 & 3, computed as the sum of the inherent error and the estimated sampling error of SPC, with the total error of Method 4, which is unaffected by the sampling error

If SPC is considered the reference standard, then the achievable accuracy is limited by the variability between different human observers. To assess the feasibility of further improvements, we have compared the error of Method 4 at 20× resolution with the errors of Observer 2 & 3. The evaluation was, again, based on point scores. The results are summarized in Table 3 and in Fig. 6 (top row).

**Table 3:** Observer and sampling error evaluation results

Method	Mean Absolute Error low-grade	Mean Absolute Error high-grade
Method 4 20×	0.011 (±0.011)	0.036 (±0.026)
Observer 2	0.010 (±0.011)	0.068 (±0.030)
Observer 2 TE	0.013 (±0.012)	0.066 (±0.036)
Observer 3	0.008 (±0.008)	0.024 (±0.017)
Observer 3 TE	0.010 (±0.010)	0.029 (±0.021)

Mean absolute errors are given in area fraction (± std. dev)

**Fig. 7:**
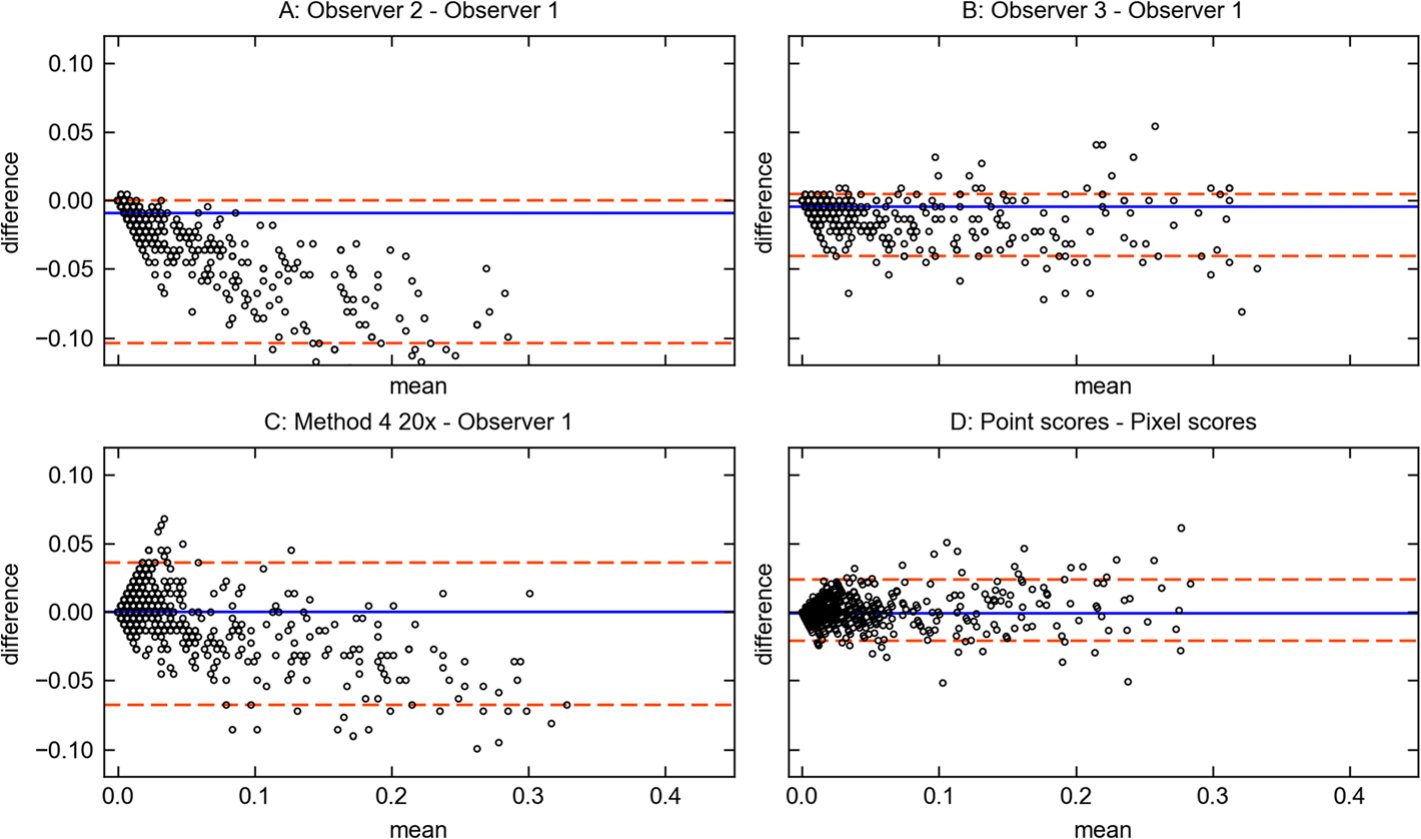
Bland-Altman plots. **a-c**: Agreement with Observer 1 of Method 4 and Observer 2 & 3. **d**: Agreement between point scores and pixel scores of Method 4 20×

On low-grade images, the error was generally low and Method 4 performed on par with the human observers. On high-grade images, the error was notably higher and more variable. Here, Method 4 was closer to the reference result than Observer 2, however, not as close as Observer 3. Figure 7a–c visualizes the respective agreement with Observer 1 as Bland-Altman plots. It becomes apparent that there was a general bias towards underestimation of the steatosis scores. For Observer 2 and Method 4, this bias increased with the magnitude of the values.

**Fig. 8:**
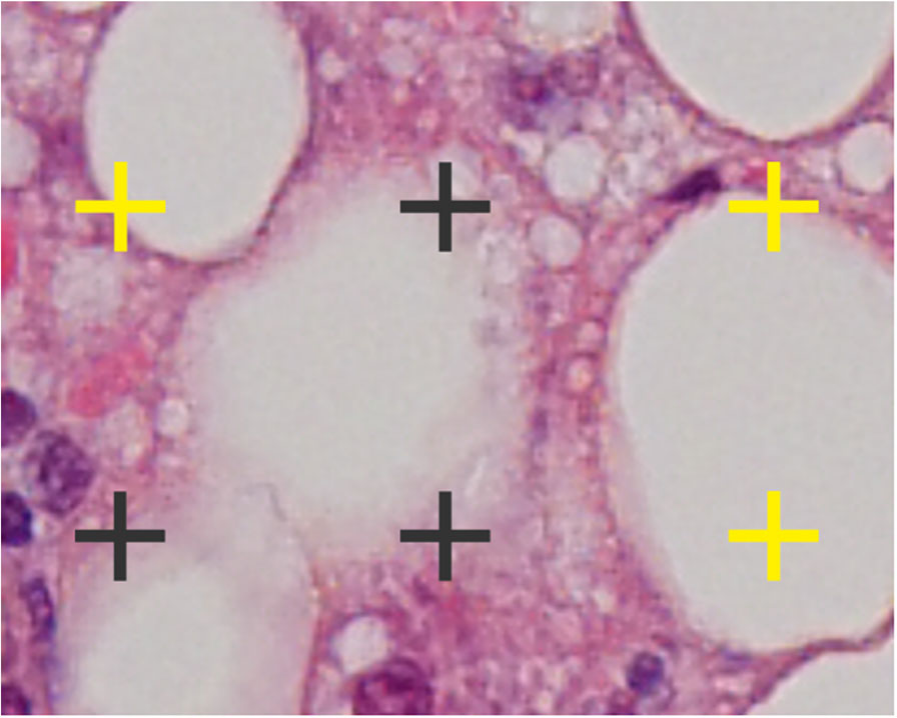
Edge Ambiguity. Because of the thickness of tissue sections, it is often ambiguous whether points lie within or outside of fat droplets

Upon visual inspection, it appeared, that neither of the observers was systematically wrong, but that there was often ambiguity in the classification of grid points. The thickness of histological sections often prevents fat droplets from being clearly delineated from tissue. Instead, there is a gradient region, where tissue slowly fades into empty space. Since the area of circular fat droplets grows quadratically with their radius, a large portion of their area is concentrated near the edge. Stereological grid points are therefore very likely to lie in the gradient region. It turns out that much of the disagreement was caused by observers being more or less restrictive in the assignment of grid points in gradient regions to fat droplets (see Fig. 8).

### Sampling error evaluation

Stereological point counting considers only a subsample of all image pixels, which naturally leads to some sampling error. In the previous sections, the inherent errors of different analysis methods and human observers were evaluated on the basis of point scores. Here, the sampling error was irrelevant because all steatosis scores were computed on the same grid of points.

Automated analysis methods are generally unaffected by the sampling error of SPC because they consider all image pixels. To take this advantage into account, we have estimated how the sampling error affects the total error of the human observers.

Since the true sampling error of the human observers was unknown, we have made the rough assumption that their sampling error equals the one of Method 4 at 20× resolution. The sampling error of Method 4 could be easily computed by subtracting its pixel scores from its point scores. The resulting sampling error distribution is visualized in Fig. 7d. It turned out to be distributed symmetrically around 0 with its variance growing in proportion to the magnitude of values. The mean absolute sampling error amounted to 0.006 (±0.006) for low grade and 0.015 (±0.012) for high-grade images.

The total error of the human observers equals their inherent error plus the sampling error of SPC. By adding the estimated sampling error of every image to the respective point scores of Observer 2 & 3, we have obtained two estimates of their total error, denoted as Observer 2 TE and Observer 3 TE. Both were compared to the inherent error of Method 4 at 20×, under the assumption that the inherent error of its point scores is a good approximation for the inherent error of its pixel scores. The results are summarized in Table 3 and in Fig. 6 (bottom row).

The added sampling error caused a small but noticeable increase in the total error of Observer 2 & 3. Since the sampling error can be positive or negative it can increase or decrease the total error on individual images. This explains the different effect on both observers. When the sampling error was taken into account, the total error of Method 4 was close to that of Observer 3. However, it still was higher on both low-grade and high-grade images.

## Discussion

We have evaluated different automated image analysis methods for quantifying steatosis in terms of their agreement with SPC performed by a hepatologist. In this context, we have presented a new method that intends to improve the robustness to image variability. While most methods had different merits for low-grade and high-grade images, the new method achieved the best agreement on both image groups.

The error of the new analysis method decreased when it was applied to higher image resolutions. However, this decrease came at the expense of runtime. Savings in runtime are important when analyzing large whole slide images. In this case, the slightly larger error at 10× resolution can be more acceptable than the significantly higher runtime at 20× resolution.

We have compared the new analysis method with SPC performed by two additional human observers, who were knowledgeable in the task and experienced in the quantitative analysis of medical images. It turned out that the error of the analysis method was substantially lower than one observer and only slightly above the other observer. This applied especially when the sampling error of SPC was taken into account. The results suggest that the new method can be a suitable automated replacement for SPC.

We have also seen that there is considerable variability between human observers, partly because of general ambiguity about the edges of fat droplets due to the thickness of tissue sections. Further reductions in error, therefore, do not necessarily indicate actual improvements, but can indicate over-fitting towards a specific observer.

One limitation of the work is that, because of the high effort of SPC, the evaluation was performed against only one expert. Future work should be carried out to assess the variability of SPC between multiple experts. Only if automated methods are evaluated against this variability, it will be possible to verify further improvements.

Another limitation is that the evaluation was performed without consideration of the size of fat droplets. The size distribution of fat droplets may hold important diagnostic information [8, 14]. Therefore, it will also be important to evaluate the agreement between human observers and automated analysis methods with respect to droplet sizes.

## Conclusions

Automated image analysis methods for quantifying steatosis should be evaluated against other area-based measurement, such as SPC. We have presented a new method that achieved the best agreement with SPC performed by a hepatologist. The method is simple to implement and showed a good trade-off between accuracy and runtime. A comparison with additional human observers suggested that the new method can be a suitable automated replacement for SPC. Before further improvements can be verified, it is necessary to thoroughly assess the variability of SPC between human observers.

## Abbreviations

AFLD: Alcoholic fatty liver disease
FLD: Fatty liver disease
HE: Hematoxylin and Eosin
NAFLD: Nonalcoholic fatty liver disease
SPC: Stereological point counting
